# Nutritional Interventions to Improve Sleep in Team-Sport Athletes: A Narrative Review

**DOI:** 10.3390/nu13051586

**Published:** 2021-05-10

**Authors:** Madeleine Gratwicke, Kathleen H. Miles, David B. Pyne, Kate L. Pumpa, Brad Clark

**Affiliations:** 1 Research Institute for Sport and Exercise, University of Canberra, Canberra 2617, Australia; maddy.gratwicke@canberra.edu.au (M.G.); kathleen.miles@canberra.edu.au (K.H.M.); David.Pyne@canberra.edu.au (D.B.P.); Kate.Pumpa@canberra.edu.au (K.L.P.); 2 Discipline of Sport and Exercise Science, University of Canberra, Canberra 2617, Australia

**Keywords:** sleep, athletes, recovery, team-sport, macronutrients, supplements

## Abstract

Athletes often experience sleep disturbances and poor sleep as a consequence of extended travel, the timing of training and competition (i.e., early morning or evening), and muscle soreness. Nutrition plays a vital role in sports performance and recovery, and a variety of foods, beverages, and supplements purportedly have the capacity to improve sleep quality and quantity. Here, we review and discuss relevant studies regarding nutrition, foods, supplements, and beverages that may improve sleep quality and quantity. Our narrative review was supported by a semi-systematic approach to article searching, and specific inclusion and exclusion criteria, such that articles reviewed were relevant to athletes and sporting environments. Six databases—PubMed, Scopus, CINAHL, EMBASE, SPORTDiscus, and Google Scholar—were searched for initial studies of interest from inception to November 2020. Given the paucity of sleep nutrition research in the athlete population, we expanded our inclusion criteria to include studies that reported the outcomes of nutritional interventions to improve sleep in otherwise healthy adults. Carbohydrate ingestion to improve sleep parameters is inconclusive, although high glycemic index foods appear to have small benefits. Tart cherry juice can promote sleep quantity, herbal supplements can enhance sleep quality, while kiwifruit and protein interventions have been shown to improve both sleep quality and quantity. Nutritional interventions are an effective way to improve sleep quality and quantity, although further research is needed to determine the appropriate dose, source, and timing in relation to training, travel, and competition requirements.

## 1. Introduction

High-performance team-sport athletes are required to train and compete to a high standard on a weekly basis. However, these athletes face the continual challenge of balancing the physiological, neuromuscular, and psychological stressors induced by training and competition [1]. Optimizing recovery to overcome these challenges and support performance is of utmost importance [2]. Sleep plays a crucial role in recovery and has restorative effects on physiological [3], perceptual [3,4,5], and immune [6,7] functions. Athletes, regardless of their sport, rate sleep as their most important recovery strategy [8,9]. A reduction in sleep quality has been associated with increased incidence of fatigue-related injury [1], reductions in the skeletal muscle remodeling [10], and disturbs cellular maintenance processes [6,7]. Despite evidence affirming the importance of restorative sleep [11], elite athletes appear to experience more sleep disturbances than the general population [1,8]. Behavioral and lifestyle strategies that promote sleep and sports performance will be welcomed by athletes and coaches.

Research examining athlete sleep indicates evening competition, travel, and training schedules can have a deleterious impact on sleep quality and quantity [12]. Up to ~50% of elite athletes experience sleep disturbances following late night competition or training sessions [1,13]. Night-time competition is widely associated with sleep issues and athletes can experience substantial disruptions (i.e., increased core temperature, muscle soreness, and delayed bedtime) to sleep quality and quantity compared with daytime competition [12]. Athletes also often face undesirable training schedules, including morning or late evening sessions, which can compromise an optimal recovery regime by reducing sleep quantity due to limited time in bed [10,12,14]. Athlete sleep quantity is also impacted by air travel, which can reduce sleep duration given a later sleep onset, delayed time in bed [15], and circadian rhythm misalignment [12]. A variety of interventions have been explored to negate the disturbances experienced by athletes, although not all have proven to be successful.

Evidence for the efficacy of interventions to improve sleep and related recovery outcomes in athletes is somewhat equivocal. Research investigating the effect of a sleep hygiene intervention in professional rugby league indicated sleep duration and efficiency increased immediately following the education seminars, but a follow-up one month later saw sleep behavior comparable to baseline levels [16]. Sleep hygiene and sleep extension interventions require athletes to change lifestyle behaviors (not always practical around training and competition schedules), which may yield poor compliance and limited effectiveness without regular follow-up [16]. Interventions that enhance athlete sleep and recovery regardless of situational changes (e.g., training times or travel demands) require further evaluation including both efficacy and practical means of implementation. Both sleep hygiene education and sleep extension interventions are effective short term, however long-term sustainability of their effects is questionable [17]. Furthermore, a recent survey study indicated female athletes may be resistant or less likely to implement sleep hygiene interventions [18]. Easily implemented techniques and strategies to improve sleep in the female athlete population are required.

It is well established that nutrition plays a vital role in performance and recovery; however, research investigating the role of selected foods, and macro- and micro-nutrients in the sleep–wake cycle is in its infancy. While the primary role of sports nutrition has been to support intensive training requirements and promote recovery [19], attention is shifting to the use of nutritional supplements for improving sleep [10]. Therefore, the aim of this review was to investigate nutritional strategies that can be utilized to enhance sleep quality and quantity, and how this information can guide future nutritional interventions focused on supporting sleep in an athletic population.

## 2. Methodology

A narrative review was conducted between March and November 2020. The narrative review was supported by a semi-systematic approach to article searching, and specific inclusion and exclusion criteria, so that articles reviewed were relevant to athletes and sporting environments. Initial studies of interest were located through a search of six databases (PubMed, Scopus, CINAHL, EMBASE, SPORTDiscus, and Google Scholar) from inception up to November 2020. The following search terms were used: nutrition OR nutritional intervention AND sleep AND athletes OR athlete OR team-sport OR sportsman OR sportswoman. Another search was conducted using the following search terms: nutrition OR nutritional intervention AND sleep, to find interventions based on other populations.

Studies were included in the review if they were experimental, including randomized controlled trials, observation studies, case studies, and case reports conducted in elite or semi-elite athlete cohorts. Given the paucity of sleep nutrition research in the athlete population, we expanded our inclusion criteria to include studies that reported the outcomes of nutritional interventions to improve sleep in otherwise healthy adults. Studies were excluded if they included participants with concussions, included participants with other disorders unrelated to sleep, participants in shift work, described longitudinal dietary adjustment (e.g., low carbohydrate dieting), or an animal study. Reference lists of selected articles were also inspected to ensure all relevant literature was captured for review. The outcomes of nutritional intervention on sleep were detailed in four variables following existing recommendations [20]:

1. Total sleep time (TST, min)—total minutes of night-time sleep;
2. Sleep efficiency (SE, %)—the ratio of TST to time spent in bed;
3. Sleep onset latency (SOL, min)—the time between bedtime and sleep onset;
4. Wake after sleep onset (WASO, min)—the time awake after initial sleep onset but before the final awakening.

Given this study was a review, no ethics approval was required. Narrative synthesis was used to investigate and interpret both similarities and differences between studies identified with the two systematic searches, and other secondary sources deemed relevant by the research team.

## 3. Results

### 3.1. Included Studies and Characteristics

Evidence for the efficacy of nutritional interventions to improve sleep in human studies is limited, with even less research reporting their effectiveness in an athletic population. The results from the literature search for studies using athletic (*n* = 4) and both healthy (*n* = 6) and poor sleeping (*n* = 10) general population cohorts are displayed below in Table 1.

Athlete studies were in mixed sport disciplines, two in cycling [21,22] and another in basketball [23]. One study did not specify the discipline the athletes participated in [24]. Three athlete studies utilized wrist actigraphy and one the Pittsburgh Sleep Quality Index (PSQI). Healthy general population studies were predominantly in male participants (*n* = 5), with one study utilizing a male and female population. Three studies used PSG to measure sleep following nutritional intervention, and two used wrist actigraphy. Poor sleeping general population studies implemented a mixture of sleep methods, from subjective sleep questionnaires to PSG. Study participants were classified as general population (poor sleepers) if they reported any of the following: subjective sleep problems; insomnia; diagnosed sleep pathology; and a PSQI score above the threshold for a ‘sleep problem’ (i.e., 5.0 or 6.0 depending on the specific study).

Details of the nutritional interventions, as well as the impact these interventions on sleep outcomes are outlined in Table 2. These outcomes are detailed below in the categories of carbohydrates, protein, tart-cherry juice, and other interventions.

### 3.2. Carbohydrates

The majority (*n* = 3) of the identified carbohydrate nutritional intervention studies investigated the effect of high glycemic index (GI) carbohydrate consumption pre-sleep. Overall, high GI carbohydrate consumption resulted in increases in TST (7.9–62.4 min) and SE (0.4–8.1%), and consistent reductions in SOL (5.6–18.9 min). Afaghi and colleagues [26] also investigated the effect of a very low carbohydrate diet on sleep, showing an increase in TST (22.7 min) and SE (3.3%), and reduced SOL (5.6 min).

### 3.3. Protein

Two studies explored the effects of protein to improve sleep. Both studies used the whey protein isolate α-lactalbumin (dose range 20–40 g) [24,28]. In one study with healthy general populations, pre-sleep protein supplementation increased TST by 55 min, alongside a 7% increase in SE [28]. In the study by MacInnis et al. [24], α-lactalbumin had no effect on sleep variables in a small sample of cyclists (*n* = 6).

### 3.4. Tart Cherry Juice

Interventions utilizing tart cherry juice resulted in a universal increase across studies in TST (range from 29–39 min). The studies also showed modest improvements in SE (2.7–3.7%), and slight reductions in SOL (3.6–9.1 min) and WASO (16.8 min). The tart cherry juice studies were all performed in general population cohorts (both healthy and poor sleepers).

### 3.5. Other Interventions

The combination of γ-aminobutyric acid (GABA; an amino acid produced by natural fermentation) and *Apocynum venetum* leaf extract treatment yielded a modest decrease in SOL of 4.3 min and shortening of deep non-REM sleep latency by 5.3 min [33]. Similar magnitude reductions in SOL were reported for 100 mg of GABA supplementation only [39], while supplementation with 300 mg of GABA induced reductions in SOL and WASO in concert with an increase in SE [34]. In comparison, the consumption of two kiwifruits 1 h before bedtime yielded a substantial increase in TST of 55 min, as well as a 2% increase in SE and decreased SOL (14 min) and WASO (6.1 min) [32]. In athletes, beetroot juice showed an improvement in subjective quality of sleep as measured by the PSQI [23]. In healthy adults, 3 g of glycine ingested an hour before bed decreased SOL and the onset latency of slow wave sleep [37]. Similarly, 3 g of glycine led to decreases in morning fatigue when ingested an hour before bed [36], and an improvement in cognitive function during periods of sleep restriction when ingested 30 min before bed [29]. In adults who were dissatisfied with sleep, 3 g of L-serine ingested 30 min before bed improved subjective sleep quality and sleep satisfaction; however, in a smaller sub-study, there were no improvements in actigraphy-measured sleep [38]. Finally, consuming a tart cherry-based sleep supplement powder reduced SOL (10 min) in poor sleeping young adults [35].

## 4. Discussion

This review identified just four studies on nutritional interventions designed to enhance sleep quality and quantity in an athletic population. However, there are studies conducted in other general population cohorts, including individuals with diagnosed or self-reported sleep problems, that show modest support for the efficacy of nutritional intervention to increase objective TST, objective SE, subjective sleep, and reduce the effects of sleep complaints such as insomnia. Substantially more work is required in carefully controlled nutritional supplement studies to verify their efficacy and effectiveness in promoting sleep, recovery, and performance in the variety of settings that competitive athletes have to manage. Translation and implementation of positive experimental outcomes will require coordination and management between athletes, coaches, and support staff, especially dietitians and sports medicine practitioners, providing specific dietary advice.

### 4.1. Carbohydrate

Carbohydrates have long been utilized in sport to fuel athletes who engage in intensified and prolonged exercise [2,10]. The traditional focus of carbohydrate supplementation is often on restoring muscle and liver glycogen levels between training sessions or matches [40]. While evidence for the amount, type, and timing of carbohydrate intake for recovery is well documented, some studies have also explored the use of carbohydrate for promoting sleep.

Most studies have focused on the effects of high and low glycemic index (GI) carbohydrate feeding [22,30]. Evidence from studies in athlete samples is limited and equivocal, with one study in Brazilian male basketballers reporting a non-significant increase in TST and decrease in SOL, but also reductions in SE and an increase in WASO [22]. However, studies in healthy populations are more definitive, with consistent reports of improvements in sleep following high GI carbohydrate feeding [25,30]. In a study of particular relevance to athletes, Vlahoyiannis and colleagues [30] provided healthy, physically active young males with either a high or low GI meal immediately following an evening bout of intermittent sprint exercise. While there were no effects of meal GI on sleep architecture (i.e., proportion of sleep time spent in different sleep stages), the high GI meal substantially improved TST, SE, SOL, and WASO. As such, high GI feeding may ameliorate the sleep disturbances experienced by athletes following evening training and competition.

The timing of a high GI meal may influence its subsequent impact on sleep. One study reported that SOL was longer and subjective sleepiness was lower when a high GI meal was ingested 1 h before bed rather than 4 h before bed [25]. The proposed mechanism for improvement in sleep following a high GI meal is an increase in the plasma ratio of tryptophan to large neutral amino acids (TRP/LNAA). Tryptophan is an essential amino acid and serves as a precursor to the synthesis of serotonin and melatonin [41], both important regulators of sleep. By increasing the TRP/LNAA, tryptophan can more readily cross the blood brain barrier, consequently leading to an increase in the synthesis of serotonin [42] and then downstream increases in secretion of melatonin [25,30]. Importantly, it appears that the TRP/LNAA peaks 2–4 h after ingestion of a high carbohydrate meal with minimal change in the first 1–2 h [43]. Therefore, high GI feeding should occur at least two hours before bedtime to maximize the potential impact on sleep.

In addition to manipulating glycemic load (which combines both the quality (GI) and the quantity of carbohydrates), studies have investigated the effects of restricting carbohydrate intake on sleep parameters [26]. Ingestion of a very low carbohydrate meal 4 h before bed increased the proportion of slow-wave sleep assessed by polysomnography (PSG), the gold standard of sleep assessment [26]. An increase in slow-wave sleep could be particularly beneficial to athletes as it is thought to play an important role in performance recovery [44] and assists in energy conservation and recuperation of the nervous system [1,45]. However, athletes also need to consider the impact of low carbohydrate feeding on muscle and liver glycogen restoration, and decisions on priorities for refueling versus sleep promotion will need to be made.

The effects of different evening carbohydrate meals on sleep remains somewhat unclear given the heterogeneity of current research findings. Further research targeting the ideal timing, quantity, and source of carbohydrate ingestion, as well as its interaction with other macro and micronutrients is needed in an athletic population to determine its efficacy. More definitive guidelines on carbohydrate feeding to improve athlete sleep can then be developed.

### 4.2. Protein

Protein plays an important multifactorial role in an athlete’s training and competition cycle by facilitating muscle repair and remodeling and supporting adequate immune function [2,46,47]. Whey protein supplementation post-exercise and pre-sleep has been introduced to enhance whole body protein synthesis and muscle performance during overnight recovery [47]. A specific whey protein which has recently been investigated as a nutritional pre-sleep intervention is α-lactalbumin [42,48]. α-Lactalbumin is reported to have the highest natural level of tryptophan in protein food sources [48]. Tryptophan is an essential amino acid and serves as a precursor to the synthesis of serotonin and melatonin, both of which are involved in the regulation of sleep [41].

The initial evidence demonstrating the potential effectiveness of α-lactalbumin on sleep was obtained from a series of studies conducted in stress-vulnerable participants with and without sleep complaints [49,50]. Although not directly investigating sleep parameters, sleep quality may have improved with a substantial reduction in sleepiness, and improved attention processes the morning following the intervention. In addition, these studies observed an increase in TRP/LNAA, which is essential for serotonin synthesis in the brain. Another study also reported decreases in depressive feelings under stress in stress-vulnerable participants consuming α-lactalbumin [50]. There are strong links between sleep and feelings of anxiety and depression, with women experiencing more insomnia complaints than males [51]. Supplementation with α-lactalbumin that can increase sleep quality and decrease depressive feelings may be beneficial during high-stress team-sport seasons, particularly for female athletes.

Recently, Ong and colleagues investigated the efficacy of an α-lactalbumin treatment (20 g, 1 h prior to bed) in healthy male subjects with no known sleep conditions or impairments [28]. Sleep quantity was increased as was objective (13%; via actigraphy-based assessment) and subjective (11%; via sleep diary) TST compared to placebo, as well as 7% higher objective SE [28]. In comparison, there was no difference for any actigraphy-recorded sleep variables between α-lactalbumin and collagen peptide supplementation in a small cohort of cyclists [24]. While there is preliminary evidence to support the efficacy of α-lactalbumin to improve sleep, further well-designed studies are required to confirm its effectiveness. Specifically, research examining the use of α-lactalbumin chronically in an athletic population is warranted. To date, limited studies involving night-time protein ingestion have been carried out for longer than four weeks in the general population. There is currently no evidence for the efficacy of nutritional interventions when used chronically, or in a field setting in an athletic population during training and competition.

### 4.3. Tart Cherry Juice

Tart cherries contain approximately 13 ng of melatonin per kg of cherry [52], which upon consumption can increase exogenous melatonin, which is critical for the sleep–wake cycle in humans [53]. The high antioxidant content of tart cherries purportedly reduces oxidative stress, in turn enhancing sleep in isolation and in conjunction with melatonin. The reported anti-inflammatory properties may influence the pro-inflammatory cytokines involved in sleep regulation and also the recovery process after exercise [10,52]. The majority of studies investigating tart cherry juice usage in an athletic population have examined its effect on aspects of recovery including muscle soreness [54,55,56]. While promoting recovery is beneficial to athletes, tart cherry juice has enhanced sleep indices as assessed by PSG and wrist actigraphy monitoring in healthy individuals without the presence of sleep problems [31], and individuals with sleep problems such as insomnia [27]. Other studies have demonstrated that tart cherry juice can increase TST and SE, regardless of the differences in participants (good sleepers compared to individuals with insomnia) [27,57]. Consumption of a liquid blend consisting of Montmorency tart cherries and apple juice improved sleep in older adults with chronic insomnia by decreasing WASO, and reducing their insomnia questionnaire score compared to baseline [27].

The efficacy of tart cherry juice as an intervention to improve indices of recovery in marathon runners has been investigated [58]. Consumption of a tart cherry juice blend taken in the morning and afternoon (~240 mL) elicited a more rapid return of strength post-race and smaller elevations in inflammation markers (i.e., c-reactive protein, interleukin-6, and uric acid) compared to placebo [58]. It appears that tart cherry juice may be effective at accelerating the recovery process and restoring strength, even after high-intensity endurance exercise. However, there are claims that the use of antioxidants in an athletic population is questionable given potential blunting of the training adaptation response, and reduced training efficiency [59]. One authoritative consensus group indicated that tart cherry juice interventions in athletes is not recommend nor endorsed [60]. Further research is needed to assess the use of tart cherry juice in an athletic population, including its effects on sleep and physiological recovery.

### 4.4. Other Nutritional Strategies

There are a variety of other nutritional interventions that have been explored for their ability to improve sleep in poor sleepers, including kiwifruit and herbal supplements. Kiwifruits contain a range of nutrients that potentially augment sleep and recovery [44], including serotonin, a known sleep promoting hormone that helps regulate REM sleep [61]. Improved sleep was reported in poor sleepers who ingested two kiwifruits an hour before bed over a four-week intervention period [32]. Marked increases in wrist actigraphy monitored TST (16.9%) and SE (2.4%) were evident, while subjective sleep diary recordings showed a substantial decrease in WASO and SOL. The improved sleep quality may be attributable to high levels of folate in kiwifruit [10,32]. Folate deficiency has been linked to insomnia and restless leg syndrome, both of which cause large sleep disruptions and can hinder the restorative quality of sleep [32,62].

Akin to kiwifruit consumption, ingestion of GABA in different quantities has yielded improvements in the sleep quality of poor sleepers or those dissatisfied with sleep [33,39]. GABA is an inhibitory neurotransmitter that is often present in food, and its receptors in the central nervous system are often targeted by pharmacological agents such as benzodiazepines for treatment of several conditions including insomnia [63]. While benzodiazepines can improve sleep quality and quantity, they are also associated with substantial side-effects, including drowsiness, lethargy, fatigue, and, in extreme cases, impaired motor coordination and addiction [63], all of which would likely impair athletic performance. Importantly, studies in humans have reported no adverse effects of pre-sleep GABA ingestion on next-day sleepiness or fatigue, which suggests it may be useful in athletes to improve sleep.

Glycine is another inhibitory neurotransmitter that has been linked to improvements in subjective and objective sleep quality for poor sleepers [36,37], but also daytime fatigue and cognitive performance in healthy adults during simulated sleep restriction [29]. Similarly, ingestion of L-serine, a glycine precursor, reportedly leads to improvements in sleep satisfaction and subjective sleep quality in adults dissatisfied with their sleep [38]. While further evidence for the efficacy of glycine and L-serine in athlete populations is required, these results suggest both proteins may offer athletes dissatisfied with sleep issues, or facing situational sleep restriction, a means by which to improve their sleep.

Most studies included in this review have focused on the effects of single nutrients or ingredients. However, some recent studies have examined the effects of multi-nutrient supplements on sleep. In one study on the effects of a tart cherry powder-based supplement, there was a significant improvement in SOL and a tendency towards improvement in SE across seven days of supplementation in young adult poor sleepers [35]. However, chemical analysis undertaken as part of the study indicated the supplement contained no melatonin, despite being tart cherry based. Instead, the sleep improvement was likely due to the 3 g of tryptophan and 2 g of glycine, which is similar to the 3 g dose used in other studies that report sleep improvement [29,36,37], contained in the product. Another multi-nutrient sleep study determined both the most and least optimal combination of a variety of ingredients for sleep improvement [64]. Both combinations contained a mixture of ingredients linked to improvements in sleep when used in isolation including high GI carbohydrate and tryptophan. The most optimal combination led to a reduction in SOL in a group of healthy adults with good sleep. Perhaps more importantly for athletes, the least optimal combination drink led to increases in SOL relative to a placebo supplement in the same group, emphasizing the need to consider how nutrients interact to influence sleep.

## 5. Future Directions

There are numerous nutrients and foods that have demonstrated efficacy in isolation for enhancing sleep quality and quantity. As humans rarely consume single nutrients in isolation, and typically enjoy a variety of nutrients as part of mixed meals, it is important to clarify how the co-ingestion of food and supplements can help or hinder sleep. This information would not only be beneficial for athletes within the daily training environment, but also when traveling and crossing multiple time zones with national and international travel.

The growing interest in monitoring athlete sleep has led to an increase in commercial sleep technology (wearables such as Fitbit™ and Whoop™, nearables such as ResMed S+™, and smartphone applications). These consumer-based technologies have advantages in terms of low-cost and ease of use, however the reliability and validity of many of these devices remains to be established. Although caution needs to be taken when implementing these commercial sleep technology devices at present, the market for sleep monitoring technology is expanding rapidly. Ideally, this expansion will occur alongside research on the reliability and validity of these devices for use in applied settings.

With a plethora of supplements now available in different forms (powder, liquid, capsule), investigation is also required on the most effective form(s) of supplement to consume. For example, tart cherry is available as a liquid, powder, and in a capsule. Understanding the most effective dosage (for example g per kg body mass), timing (acute or chronic consumption), and the most effective supplement form (liquid, gel, powder, capsule), would enable practitioners to more accurately individualize and prescribe nutritional interventions and supplements to aid sleep. More work is needed to establish optimal nutrient dosing relative to body mass, rather than absolute dosing (fixed amount(s) of nutrients) used in the majority of existing studies.

## 6. Practical Applications

Given the well-known benefits of sleep for athletes, other than implementing appropriate sleep hygiene practices such as creating a sleep routine, avoiding electronic devices, and sleeping in a cool, dark quiet room, nutritional interventions can be useful in enhancing sleep quality and quantity. Though the exact timing and dosage of nutrition interventions to enhance sleep warrants further research, the following sleep enhancement strategies can be derived from the studies published to date:

- Consume a diet rich in fiber, whole grains, fruits, and vegetables.
- Consume a high GI carbohydrate meal 2–4 h before bedtime.
- Incorporate tart cherry juice concentrate into an athlete’s daily routine when sleep may be impaired (e.g., night competition), 1 × 30 mL upon waking and 1 × 30 mL before the evening meal.
- Consume 20–40 g of a protein source rich in tryptophan, such as α-lactalbumin enriched whey protein, 2 h before bedtime.
- Regular kiwifruit consumption an hour before bed.
- Glycine at a dosage of 3 g consumed before bed may enhance sleep quality and quantity.

To identify if a nutritional intervention is enhancing the sleep of an individual athlete in an applied setting, we recommend:

- Using a reliable and valid device such as wrist actigraphy, and questionnaires that assess sleep quality (i.e., Pittsburgh Sleep Quality Index) [65] and quantify daytime sleepiness (Epworth Sleepiness Scale) [66].
- Considering alternative options available to monitor sleep depending on access to expertise, the simplicity of use, and reliability and validity of the method [67].

In relation to nutritional interventions known to compromise sleep, we recommend that athletes avoid the following substances prior to sleep:

- Caffeine [68].
- Alcohol [69].
- Excess fluid ingestion [70].

A major challenge for athletes is to maintain high standard nutritional practices across the wide variety of settings they face: their personal home environment, the regular training facility, the home match venue, the away match venue, and during local, national, and international travel. Dieticians should work closely with and educate players, coaches, and team staff to ensure that both nutrition and sleep arrangements are given a high priority.

## 7. Conclusions

It appears that selected nutritional interventions are an effective way to improve sleep quality and quantity, if the correct dose, source, and timing is administered. However, the underlying efficacy and real-world effectiveness of these nutritional interventions in elite athletes is unclear, given the limited number of studies conducted in this cohort. Given that sleep is acknowledged as an important factor during training, travel, and competition, future research needs to assess the ability of these nutritional interventions to support athlete sleep for both training and competition.

## Figures and Tables

**Table 1:** Characteristics of studies in athlete, and healthy and poor sleeping general population groups.

Study	Subjects			Method of Sleep Assessment
	Description	*n*	Age, y	
**Athletes**				
Killer, 2017 [21]	Trained male cyclists	10	25.0 ± 5.8	Wrist actigraphy
Daniel, 2019 [22]	State-level male basketball players	8	18.0 ± 0.7	Wrist actigraphy
Shamloo, 2019 [23]	Male athletes	30	20.7 ± 3.7	PSQI
MacInnis, 2020 [24]	Elite male and female track cyclists	6	23 ± 6	Wrist actigraphy
**General Population (Healthy)**				
Afaghi, 2007 [25]	Healthy males	12	18–35	PSG
Afaghi, 2008 [26]	Health, non-obese males	14	18–35	PSG
Howatson, 2012 [27]	Healthy and physically active males and females	20	26.6 ± 4.6	Wrist actigraphy
Ong, 2017 [28]	Healthy males	10	26.9 ± 5.3	Wrist actigraphy
Bannai, 2012 [29]	Healthy males	7	40.6 (30–61)	Subjective sleep questionnaires
Vlahoyiannis, 2018 [30]	Healthy males	10	23.2 ± 1.8	PSG
**General Population (Poor Sleepers)**				
Pigeon, 2010 [31]	Chronic insomnia, otherwise healthy	15	71.6 ± 5.4	Subjective sleep questionnaires
Lin, 2011 [32]	Poor sleepers, males and females	24	20–55	Wrist actigraphy and PSQI
Yamatsu, 2015 [33]	Poor sleepers, males and females	16	36.8 ± 8.9	One channel EEG
Byun, 2018 [34]	Poor sleepers, males and females	30	49 ± 14	PSG and PSQI
Simper, 2019 [35]	Poor sleepers, young male and female adults	19	21.0 ± 1.0	Wrist actigraphy
Ingawa, 2006 [36]	Poor sleepers, females	15	31.1 (24–53)	Subjective sleep questionnaires
Yamadera, 2007 [37]	Poor sleepers, males and females	11	40.5 ± 10.1	PSG and subjective sleep questionnaires
Ito, 2014 [38]	Poor sleepers, males and females	45	35 ± 8	Subjective sleep questionnaires
Ito, 2014 [38]	Poor sleepers, males and females	6	35 ± 8	Wrist actigraphy
Yamatsu, 2016 [39]	Poor sleepers, males and females	10	37.7 ± 11.5	One channel EEG

EEG: electroencephalogram, PSG: polysomnography, PSQI: Pittsburgh Sleep Quality Index, data presented as mean ± SD unless otherwise noted.

**Table 2:** Changes to sleep following carbohydrate, protein, tart cherry juice, and other nutritional interventions.

Study	Intervention			TST (min)	SE (%)	SOL (min)	WASO (min)
	Type	Timing	Days				
**Carbohydrate**							
Afaghi, 2007 [25]	High GI dinner	4 h pre-bed	3	↑ 7.9	↑ 1.7	↓ 8.5*	↑ 1.7
Daniel, 2019 [24]	High GI dinner and evening snack	4 h pre-bed	1	↑ 26.5	↓ 1.2	↓ 12.5	↑ 9.0
Afaghi, 2008 [26]	Very low carbohydrate diet (<1% total energy intake)	Over day	4	↑ 22.7	↑ 3.3	↓ 5.4 *	↓ 8.6
Killer, 2017 [21]	High carbohydrate drinks	Pre-, during, and post-exercise	9	↓ 19.0*	↓ NR	⟷ NR	NR
Vlahoyiannis, 2018 [30]	High GI dinner	Post-exercise (~2 h pre-bed)	1	↑ 62.4 *	↑ 8.1 *	↓ 18.9 *	↓ 32.9 *
**Protein**							
Ong, 2017 [28]	Serving (20 g) of α-lactalbumin	1 h pre-bed	2	↑ 54.7 *	↑ 7.0 *	↓ 10.1	↓ 20.8
MacInnis, 2020 [24]	Serving (40 g) of α-lactalbumin	2 h pre-bed	3	⟷ NR	⟷ NR	⟷ NR	⟷ NR
**Tart Cherry Juice**							
Pigeon, 2010 [31]	Serving (240 mL) of tart Montmorency cherry juice	8:00–10:00 and 1–2 h pre-bed	14	↑ 29.3 **	↑ 3.7 *	↓ 3.6 **	↓ 16.8 **
Howatson, 2012 [27]	Serving (30 mL) of tart Montmorency cherry juice (with 200 mL water)	30 min post-wake and 30 min pre-bed	7	↑ 39.0 *	↑ 2.7	↓ 9.1	NR
**Other**							
Lin, 2011 [32]	Two green kiwifruits	1 h pre-bed	28	↑ 54.8 **	↑ 2.0 **	↓ 13.9 **	↓ 6.1 **
Yamatsu, 2015 [33]	GABA (100 mg) with AVLE (50 mg)	30 min pre-bed	14	NR	NR	↓ 4.3	NR
Shamloo, 2019 [23]	Serving (100 mL) of beetroot juice	2 h pre-exercise	7	NA	NA	NA	NA
Inagawa, 2006 [30]	Glycine (3 g)	1 h pre-bed	4	NA	NA	NA	NA
Yamadera, 2007 [37]	Glycine (3 g)	1 h pre-bed	2	⟷ NR	NA	↓ NR **	⟷ NR
Bannai, 2012 [29]	Glycine (3 g)	30 min pre-bed	3	NA	NA	NA	NA
Ito, 2014 [38]	L-serine (3 g)	30 min pre-bed	4	NA	NA	NA	NA
Ito, 2014 [38]	L-serine (3 g)	30 min pre-bed	2	NA	NA	NA	NA
Yamatsu, 2016 [39]	GABA (100 mg)	30 min pre-bed	7	NA	⟷ NR	↓ 5.0 *	NA
Byun, 2018 [34]	GABA (300 mg)	1 h pre-bed	28	↑ 8.6	↑ 6.7 *	↓ 7.7 **	↓ 19.6 *
Simper, 2019 [35]	Serving of “Night Time Recharge” sleep supplement	1 h pre-bed	7	↑ 0.37	↑ 5.9	↓ 10**	NR

AVLE: *Apocynum venetum* leaf extract, GABA: γ-aminobutyric acid, GI: glycemic index, GL: glycemic load, NA: not applicable, NR: value not reported, SE: sleep efficiency, SOL: sleep onset latency, TST: total sleep time, ↑ increase, ↓ decrease, ⟷ no change, * *p* < 0.05, ** *p* < 0.01.

## Data Availability

Not applicable.
